# Decreasing trends in thyroid cancer incidence in South Korea: What happened in South Korea?

**DOI:** 10.1002/cam4.3926

**Published:** 2021-05-12

**Authors:** Chang‐Mo Oh, Jiwon Lim, Yuh Seog Jung, Yeol Kim, Kyu‐Won Jung, Seri Hong, Young‐Joo Won

**Affiliations:** ^1^ Department of Preventive Medicine School of Medicine Kyung Hee University Seoul South Korea; ^2^ Division of Cancer Registration and Surveillance National Cancer Control Institute National Cancer Center Goyang South Korea; ^3^ Department of Otolaryngology‐Head and Neck Surgery Head & Neck Oncology Clinic Center for Thyroid Cancer National Cancer Center Hospital Goyang South Korea; ^4^ Department of Cancer Control and Population Health National Cancer Center Graduate School of Cancer Science and Policy National Cancer Center Goyang South Korea; ^5^ Division of Cancer Management & Policy National Cancer Control Institute National Cancer Center Goyang South Korea

**Keywords:** incidence, mortality, reservoir, South Korea, thyroid neoplasm

## Abstract

**Background:**

South Korea has the highest incidence of thyroid cancer in the world. Our study examined the trends in thyroid cancer incidence by the histologic type, cancer stage, and age group and explored possible factors that affected thyroid cancer trends.

**Methods:**

We conducted a descriptive epidemiological study using the national cancer registry data and cause of death data from 1999 to 2016 in South Korea. Age‐standardized rates were calculated using Segi's world standard population. Joinpoint regression analysis was applied to determine the changing point of thyroid cancer trends according to histologic type; Surveillance, Epidemiology, and End Results (SEER) summary stage; and age groups by sex.

**Results:**

The age‐standardized incidence of thyroid cancer in both men and women increased from 6.3 per 100,000 people in 1999 to 63.4 per 100,000 in 2012 but declined from 2012 to 2016, before the debates for over diagnosis of thyroid cancer began in 2014. The age‐standardized mortality rate of thyroid cancer, incidence of distant thyroid cancer, and incidence of regional and localized thyroid cancer started to decline since early 2000, 2010, and 2012, respectively. In addition, thyroid cancer prevalence in thyroid nodules showed decreasing trends from 1999–2000 to 2013–2014.

**Conclusions:**

The incidence of thyroid cancer began declining from 2012, before the debates for over diagnosis of thyroid cancer began in 2014. Changes in guidelines for thyroid nodule examinations may have affected this inflection point. Moreover, the debates for over diagnosis of thyroid cancer may have accelerated the decline in thyroid cancer.

## Graphical abstract

The incidence of thyroid cancer in South Korea has peaked in 2012 and started to decline thereafter. The reduction in thyroid cancer incidence was accelerated by debates for over diagnosis of thyroid cancer in 2014, but changes in the trends of thyroid cancer by stage have already been observed before 2013.

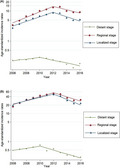

**Lay summary**

South Korea had the highest incidence of thyroid cancer worldwide in 2012, and this was primarily due to over diagnosis. However, the incidence had shown decreasing trends starting from 2013, and the reason for such sudden decline is controversial. Although improvements in over diagnosis rates was considered to be the main reason, the incidence began to decline in 2013, while concerns on over diagnosis were only raised starting in 2014. In addition, there were already signs such as a decrease in mortality and sequential decrease of distant stage of thyroid cancer before 2013.

## 1 INTRODUCTION

In most countries, the incidence of thyroid cancer show increasing trends.^1^ South Korea has an extremely high thyroid cancer incidence, which increased rapidly since 1999^2^ mostly because of the increase in thyroid nodule and thyroid cancer detection.^2^, ^3^ However, the thyroid cancer incidence in South Korea recently underwent a drastic change^4^, ^5^ and continuously decreased after the highest peak in 2012.^5^

The reason for the sudden decline in thyroid cancer incidence was controversial. It was argued that the incidence decreased because of the issue of thyroid cancer over diagnosis.^4^ On the other hand, changes in the guidelines for examination of thyroid nodule could affect the pattern of thyroid cancer detection; therefore, the thyroid cancer incidence declined due to the reduction in small‐sized thyroid cancer detection.^6^

Therefore, we investigated the time point of change in thyroid cancer incidence according to the histologic type, cancer stage, and age group by sex and explored the possible factors that affected the timing of these changes.

## 2 MATERIALS AND METHODS

### 2.1 Data source

The Korean Central Cancer Registry (KCCR) has systematically collected nationwide cancer incidence data since 1999. It currently publishes annual reports on cancer incidence, prevalence, and survival in South Korea.^5^ Moreover, it has submitted cancer incidence and survival data for five continents and the Cancer survival in five continents: a worldwide population‐based study‐3 (CONCORD‐3) study as Korea's representative cancer registration data.^7^, ^8^ Thus, the quality of data reported by the KCCR is of international standard.^7^, ^8^ Indeed, the completeness of cancer incidence data was estimated to be 98.2% according to the method proposed by Ajiki et al. The process of data collection and statistical calculations are described in detail in the annual report.^5^

In this study, we used the cancer incidence data from the KCCR between 1999 and 2016 to estimate the trends of thyroid cancer incidence in South Korea. The cause of death database from Statistics Korea from 1999–2016 was used to estimate the trends in the mortality rate of thyroid cancer. The study protocol was approved by the Institutional Review Board of Kyung Hee University (IRB number: KHSIRB‐19–201).

### 2.2 Definition and classification of thyroid cancer

Thyroid cancer was defined as “C73” according to the ICD‐10 code.^9^ The histological subtypes were classified as papillary thyroid carcinoma (morphology codes: 8050, 8260, 8340–8344, 8350, 8450–8460), follicular thyroid carcinoma (morphology codes: 8290, 8330–8335), medullary thyroid carcinoma (morphology codes: 8345, 8510–8513), anaplastic thyroid carcinoma (morphology codes: 8020–8035), and others according to the International Classification of Diseases for Oncology, 3rd edition.^10^, ^11^ Patients with thyroid cancer at cancer diagnosis were divided into 5 age groups: <20, 20–44, 45–54, 55–64, and ≥65 years. The KCCR has collected nationwide cancer staging information using the Surveillance, Epidemiology, and End Results (SEER) summary stage since 2005.^12^ SEER summary stage was used to examine the trends in the incidence of thyroid cancer by cancer stage, and the SEER summary stage was classified into localized (cancer located within the original organ and not found elsewhere), regional (cancer invading the regional lymph node or organ located around the origin, without remote metastasis), distant (cancer fallen away from the primary organ and spread to other tissues far away), and unknown stages.^12^, ^13^

### 2.3 Statistical analysis

The baseline characteristics of patients were compared by the time period of cancer diagnosis (1999–2001 to 2014–2016). Continuous variables and categorical variables are expressed as mean ±standard deviation and number (percentage), respectively. Information on treatment was classified into those who underwent surgery within 4 weeks after diagnosis and others (surgery vs. others). Differences in continuous variables for each diagnosis period were tested using ANOVA, while differences in the distribution of baseline characteristics across the categorical variables were tested using the Chi‐square test. A direct age‐standardized method was used to compare incidences by year and to estimate the Joinpoint of the trend. Age‐standardized incidences were calculated in units of 5 years (0–5 to ≥85 years old) using the Segi's world standard population as the standard population (Table S1).^14^

The Joinpoint regression model was used to detect the best‐fitting points with significant changes in the thyroid cancer incidence trend.^15^, ^16^ Annual percent changes (APCs) were used as estimators to summarize the rate of change for each interval, and the average annual percent change (AAPC) was estimated as a weighted average of APCs to summarize the overall trend for the entire period between 1999 and 2016. The Joinpoint regression model was also applied to examine the significant changes in the thyroid cancer mortality trend.

*P*‐values <0.05 were statistically significant. All statistical analyses were performed using SAS 9.4 (SAS Institute, Cary, NC, U.S.A.) and STATA software version 14 (StataCorp LP, College Station, TX, USA).

## 3 RESULTS

### 3.1 Baseline characteristics of thyroid cancer patients from 1999 to 2016

From 1999 to 2016, 392,668 patients were diagnosed with thyroid cancer in South Korea (Table 1). The mean age of patients with thyroid cancer diagnosed from 1999 to 2001 was 46.4 ± 14.9 years; the majority were aged 20–44 years (45.8%). By SEER summary stage, the percentage of localized thyroid cancer cases increased from 2005–2007 to 2011–2013, and then decreased slightly from 43.1% in 2011–2013 to 39.4% in 2014–2016. However, the percentage of regional thyroid cancer cases gradually increased from 41.1% in 2005–2007 to 52.6% in 2014–2016. The percentage of patients who did not undergo surgery within 4 months of thyroid cancer diagnosis increased from 7.8% in 2005–2007 to 9.9% in 2008–2010 and from 9.6% in 2011–2013 to 12.8% in 2014–2016.

**TABLE 1:** Baseline characteristics of thyroid cancer in South Korea by time of thyroid cancer diagnosis

Variables	1999–2001		2002–2004		2005–2007		2008–2010		2011–2013		2014–2016		*p*‐value^a^
	Cases	%	Cases	%	Cases	%	Cases	%	Cases	%	Cases	%	
Total	11023	100.0	23409	100.0	50248	100.0	96615	100.0	128909	100	82464	100.0	‐
Age at Diagnosis													
Mean (SD)	46.4 (14.9)		46.5 (13.4)		47.3 (12.3)		47.7 (12.1)		48.1 (12.2)		47.8 (12.7)		<0.001*
<20 years	227	2.06	282	1.20	326	0.65	498	0.52	617	0.48	574	0.70	<0.001*
20‐44 years	5045	45.77	10563	45.12	20428	40.65	38279	39.62	50462	39.15	33642	40.80	
45‐54 years	2440	22.14	6268	26.78	16252	32.34	31852	32.97	40536	31.45	23604	28.62	
55‐64 years	1938	17.58	3923	16.76	8510	16.94	16872	17.46	24964	19.37	16410	19.90	
≥65 years	1373	12.46	2373	10.14	4732	9.42	9114	9.43	12330	9.56	8234	9.98	
Sex													
Men	1628	14.77	3171	13.55	7308	14.54	15937	16.50	23816	18.48	17200	20.86	<0.001*
Women	9395	85.23	20238	86.45	42940	85.46	80678	83.50	105093	81.52	65264	79.14	
Histology type^b^													
Papillary	9255	83.96	20957	89.53	45877	91.3	91853	95.07	125142	97.08	79216	96.06	<0.001*
Follicular	818	7.42	1109	4.74	1305	2.60	1557	1.61	1831	1.42	1413	1.71	
Medullary	149	1.35	214	0.91	293	0.58	412	0.43	502	0.39	316	0.38	
Anaplastic	127	1.15	117	0.50	124	0.25	190	0.20	181	0.14	197	0.24	
Others	674	6.12	1012	4.31	2649	5.27	2603	2.69	1253	0.98	1322	1.60	
SEER summary Stage^c^													
Localized	‐	‐	‐	‐	21256	42.3	40992	42.43	55576	43.11	32488	39.40	<0.001*
Regional	‐	‐	‐	‐	20635	41.07	45378	46.97	64623	50.13	43377	52.60	
Distant	‐	‐	‐	‐	665	1.32	950	0.98	816	0.63	589	0.71	
Unknown	‐	‐	‐	‐	7692	15.31	9295	9.62	7894	6.12	6010	7.29	
Treatment^d^													
Surgery	9783	88.75	21396	91.40	46310	92.16	87055	90.11	116556	90.42	71874	87.16	<0.001*
Others	1240	11.25	2013	8.60	3938	7.84	9560	9.89	12353	9.58	10590	12.84	

^a^ ANOVA tests were performed to test differences between continuous variables and chi‐square tests were performed to test differences in distribution between categorical variables.

^b^ The histological subtypes of thyroid cancer were classified as papillary thyroid carcinoma, follicular thyroid carcinoma, medullary thyroid carcinoma, anaplastic thyroid carcinoma and others according to the International Classification of Diseases for Oncology, 3rd edition.

^c^ SEER summary stage was classified into localized stage, regional stage, distant stage and unknown stage and it has been collected nationally since 2006.

^d^ Information on treatment was classified into those who underwent surgery within 4 months after diagnosis and the others (Surgery vs. others).

* *p*‐value <0.05

### 3.2 Age‐standardized thyroid cancer incidence

Age‐standardized thyroid cancer incidences showed similar patterns in both men and women (Table S2‐S4). It increased steadily from 1999–2012 but abruptly dropped in 2013–2014 in both men and women. This sudden decrease in the thyroid cancer incidence was mostly due to a decrease in patients aged 45–64 years, decrease in the papillary carcinoma incidence, and decrease in the incidence of localized and regional thyroid cancer. It is noteworthy that the incidence of distant thyroid cancer has been steadily decreasing since 2007–2009, considering that the KCCR only began collecting SEER summary stage information since 2005. However, there was no remarkable change in the anaplastic thyroid cancer incidence in both the sexes from 1999–2001 to 2014–2016.

### 3.3 Joinpoint regression analysis for trends in the incidence of thyroid cancer by histologic type

Joinpoint regression analysis showed the trends in incidence of thyroid cancer in both men and women (Table 2). The sharp increase of thyroid cancer slowed down since 2009 and 2008 for men and women, respectively. For both sexes, the thyroid cancer incidence showed a decreasing trend from 2012 to 2016 (men: APC = −11.8, 95% confidence interval [CI]: −17.4 to −5.9; women: APC = −16.3, 95% CI: −21.2 to −11.1). All histologic types except anaplastic carcinoma started to decrease after 2012 in both men and women. However, there was no significant change in the anaplastic thyroid carcinoma incidence in both sexes during the overall study period.

**TABLE 2:** Joinpoint regression analysis for trends in incidence rate of thyroid cancer by histologic type

Categories	Trend 1		Trend 2		Trend 3		AAPC (95% CI)
	Years	APC (95% CI)	Years	APC (95% CI)	Years	APC (95% CI)	
Men							
Overall	1999**–**2009	26.3 (21.7 to 31.1)*	2009**–**2012	15.2 (−5.9 to 41.2)	2012**–**2016	−11.8 (−17.4 to −5.9)*	14.2 (9.8 to 18.8)*
Papillary	1999**–**2009	29.6 (24.6 to 34.7)*	2009**–**2012	16.2 (−4.7 to 41.7)	2012**–**2016	−12.1 (−17.5 to −6.3)*	16.0 (11.6 to 20.7)*
Follicular	1999**–**2012	7.8 (5.5 to 10.2)*	2012**–**2016	−9.8 (−18.3 to −0.3)	‐	‐	3.4 (0.7 to 6.1)*
Medullary	1999**–**2012	8.7 (5. to 12.4)*	2012**–**2016	−12.4 (−24.7 to 1.8)	‐	‐	3.3 (−0.7 to 7.5)
Anaplastic	1999**–**2016	0.4 (−1.5 to 2.3)	‐	‐	‐	‐	0.4 (−1.5 to 2.3)
Women							
Overall	1999‐2008	26.3 (22.0 to 30.8)*	2008**–**2012	10.9 (1.7 to 21.0)*	2012**–**2016	−16.3 (−21.2 to −11.1)*	11.2 (8.2 to 14.3)*
Papillary	1999‐2009	26.1 (22.3 to 30.0)*	2009**–**2012	9.1 (−8.6 to 30.2)	2012**–**2016	−16.2 (−21.3 to −10.7)*	11.7 (7.9 to 15.6)*
Follicular	1999‐2012	4.4 (2.6 to 6.1)*	2012**–**2016	−9.4 (−17.2 to −0.8)	‐	‐	0.9 (−1.3 to 3.2)
Medullary	1999‐2012	8.6 (5.8 to 11.4)*	2012**–**2016	−19.8 (‐30.0 to −8.2)*	‐	‐	1.1 (−2.3 to 4.6)
Anaplastic	1999‐2016	−1.4 (−3.4 to 0.7)	‐	‐	‐	‐	−1.4 (−3.4 to 0.7)

Note

The age‐standardized incidence rates are calculated as incidence cases per 100,000 people using Segi’s world standard population.

Abbreviations: 95% CI, 95% Confidence intervalAAPC, Average annual percent change; APC, Annual percent change.

* p‐value <0.05

### 3.4 Joinpoint regression analysis for trends in the thyroid cancer incidence by age group

There was a slight difference between men and women in the results of Joinpoint regression according to the age group (Table 3). The incidence of thyroid cancer among men aged <20 years showed an increasing trend steadily from 1999 to 2016, whereas the incidence among women aged <20 years started to decrease after 2013, but this was not statistically significant. For all age groups, except for men aged 45–54 years, the incidence of thyroid cancer showed a decreasing trend from 2012 to 2016. In men aged 45–54 years, the incidence declined since 2013.

**TABLE 3:** Joinpoint regression analysis for trends in incidence rate of thyroid cancer by age group

Categories	Trend 1		Trend 2		Trend 3		Trend 4		AAPC (95% CI**)**
	Years	APC (95% CI)	Years	APC (95% CI)	Years	APC (95% CI)	Years	APC (95% CI)	
Men									
<20	1999‐2016	8.8 (6.6 to 11.0)*	‐	‐	‐	‐	‐	‐	8.8 (6.6 to 11.0)*
20‐44	1999‐2012	26.0 (23.0 to 29.1)*	2012‐2016	−10.9 (−17.0 to −4.3)*	‐	‐	‐	‐	16.2 (13.6 to 18.8)*
45‐54	1999‐2009	31.5 (27.9 to 35.1)*	2009‐2013	6.8 (0.1 to 13.9)*	2013‐2016	−19.4 (−25.3 to −13.1)*	‐	‐	14.8 (12.2 to 17.4)*
55‐64	1999‐2002	8.6 (‐15.8 to 40.0)	2002‐2008	30.7 (22.3 to 39.7)*	2008‐2012	13.1 (4.4 to 22.6)*	2012‐2016	‐15.0 (‐18.9 to ‐11.0)*	10.5 (5.6 to 15.7)*
≥65	1999‐2012	14.7 (12.9 to 16.6)*	2012‐2016	−15.8 (−20.4 to −10.9)*	‐	‐	‐	‐	6.7 (4.9 to 8.5)*
Women									
<20	1999‐2013	9.2 (8.0 to 10.4)*	2013‐2016	−4.8 (−13.3 to 4.5)	‐	‐	‐	‐	6.6 (4.8 to 8.4)*
20‐44	1999‐2009	24.9 (21.5 to 28.3)*	2009‐2012	9.5 (−8.3 to 30.7)	2012‐2016	−12.2 (−17.5 to −6.6)*	‐	‐	12.3 (8.6 to 16.1)*
45‐54	1999‐2008	28.7 (23.9 to 33.6)*	2008‐2012	8.6 (−0.2 to 18.1)	2012‐2016	−19.3 (−24.1 to −14.2)*	‐	‐	10.8 (7.7 to 13.9)*
55‐64	1999‐2008	27.1 (22.4 to 32.0)*	2008‐2012	11.0 (1.3 to 21.8)*	2012‐2016	−20.6 (−25.3 to −15.6)*	‐	‐	10.2 (7.1 to 13.4)*
≥65	1999‐2009	20.5 (16.8 to 24.3)*	2009‐2012	10.9 (−9.5 to 35.8)	2012‐2016	−17.4 (−23.0 to −11.4)*	‐	‐	8.6 (4.6 to 12.9)*

Note

The age‐standardized incidence rates are calculated as incidence cases per 100,000 people using Segi’s world standard population.

Abbreviations: 95% CI, 95% Confidence intervalAAPC, Average annual percent change; APC, Annual percent change.

* p‐value <0.05

### 3.5 Joinpoint regression analysis for trends in the incidence of thyroid cancer by SEER summary stage

The trends in the incidence of thyroid cancer were analyzed for an 11‐year period from 2006 to 2016 according to the SEER summary stage (Table 4). In both men and women, the incidence of localized and regional thyroid cancer decreased from 2012, while that of distant and unknown stage thyroid cancer decreased since 2010 and 2009, respectively.

**TABLE 4:** Joinpoint regression analysis for trends in incidence rate of thyroid cancer by SEER summary stage

Categories	Trend1		Trend2		AAPC (95% CI)
	Years	APC (95% CI)	Years	APC (95% CI)	
Men					
Localized	2006‐2012	19.4 (11.4 to 27.9)*	2012‐2016	−15.7 (−24.2 to −6.3)*	3.9 (−1.0 to 8.9)
Regional	2006‐2012	23.3 (15.1 to 32.1)*	2012‐2016	−11.8 (−19.8 to −3.0)*	7.8 (3.1 to 12.8)*
Distant	2006‐2010	6.8 (−5.2 to 20.3)	2010‐2016	−10.1 (−15.7 to −4.2)*	−3.7 (−8.3 to 1.1)
Unknown	2006‐2009	14.1 (−3.9 to 35.4)	2009‐2016	−6.0 (−10.0 to −1.7)*	−0.3 (−5.0 to 4.6)
Women					
Localized	2006‐2012	14.7 (8.7 to 20.9)*	2012‐2016	−19.9 (−27.3 to −11.8)*	−0.7 (−4.6 to 3.4)
Regional	2006‐2012	17.6 (10.5 to 25.0)*	2012‐2016	−15.9 (−24.0 to −7.0)*	2.8 (−1.6 to 7.4)
Distant	2006‐2010	9.2 (−2.0 to 21.7)	2010‐2016	−15.1(−20.5 to −9.2)*	−6.1 (−10.4 to −1.5)*
Unknown	2006‐2009	7.5 (−7.6 to 25.1)	2009‐2016	−9.9 (−14.0 to −5.7)*	−5.0 (−9.1 to −0.7)*

Note

^The age‐standardized incidence rates are calculated as incidence cases per 100,000 people using Segi’s world standard population. SEER summary stage was classified into localized stage, regional stage, distant stage and unknown stage^ ^and it has been collected nationally since 2006^.

Abbreviations: 95% CI, 95% Confidence intervalAAPC, Average annual percent change; APC, Annual percent change.

* *p*‐value<0.05

### 3.6 Sequential change‐point of thyroid cancer mortality rates and incidences by SEER summary stage

**FIGURE 1:**
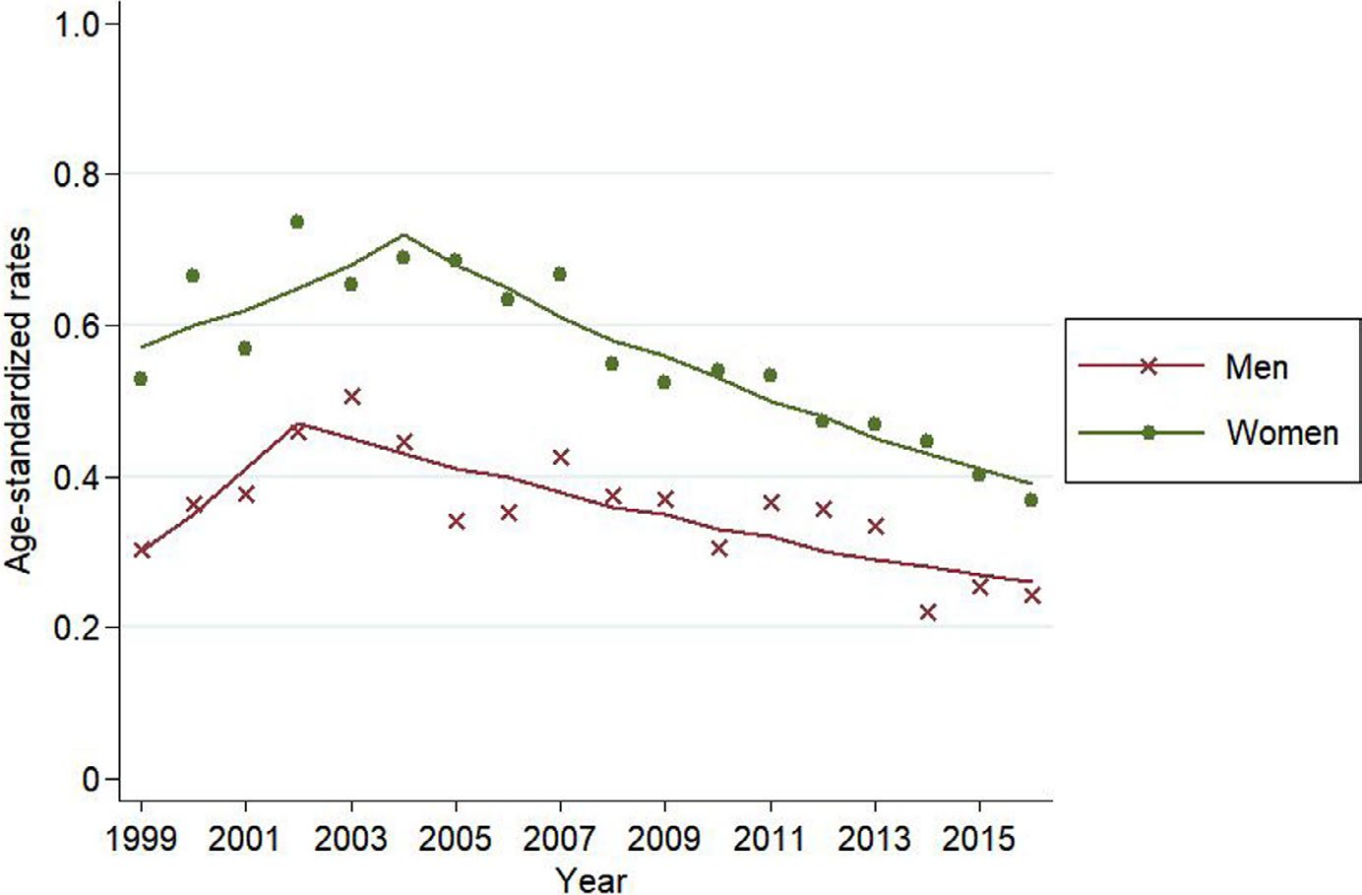
Joinpoint regression analysis for thyroid cancer mortality rates of thyroid cancer. Footnotes: The age‐standardized rates are presented as mortality cases per 100,000 people using Segi's world standard population as standard population. Joinpoint regression analysis was used to determine whether there were significant changes in trends. The lines represent the estimated trends from the joinpoint regression, and the dots represent the observed (real) rates

**FIGURE 2:**
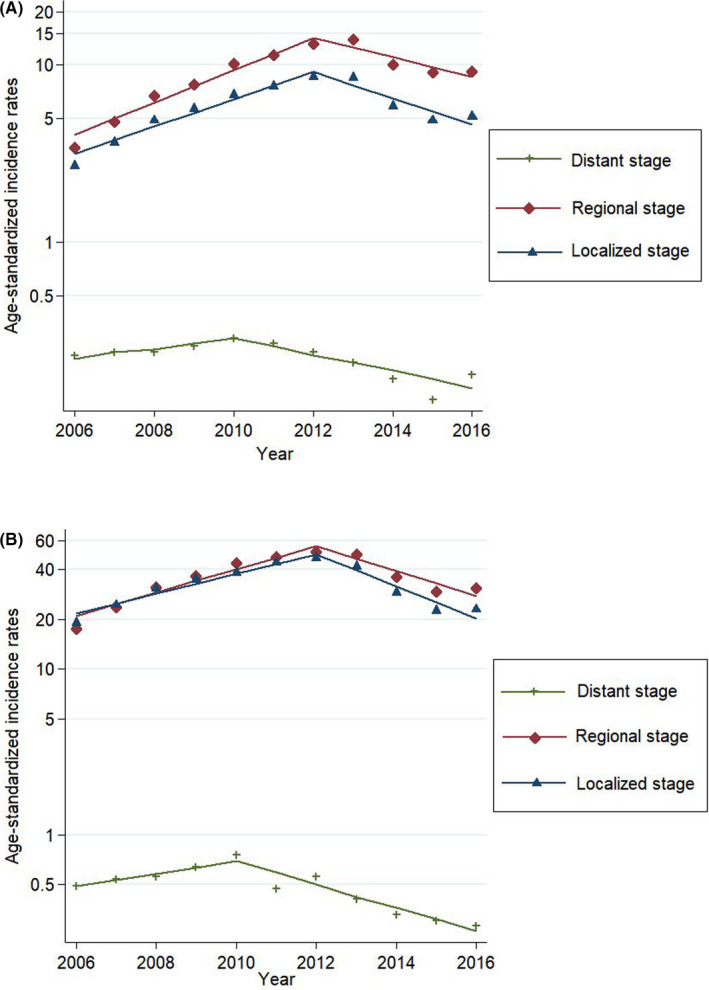
(A) Joinpoint regression analysis for thyroid cancer incidence rates of thyroid cancer by SEER summary stage in men. Footnotes: The age‐standardized rates are presented as incidence cases per 100,000 people using Segi's world standard population as standard population. Joinpoint regression analysis was used to determine whether there were significant changes in trends. The lines represent the estimated trends from the joinpoint regression, and the dots represent the observed (real) rates. (B) Joinpoint regression analysis for thyroid cancer incidence rates of thyroid cancer by SEER summary stage in women. Footnotes: The age‐standardized rates are presented as incidence cases per 100,000 people using Segi's world standard population as standard population. Joinpoint regression analysis was used to determine whether there were significant changes in trends. The lines represent the estimated trends from the joinpoint regression, and the dots represent the observed (real) rates

When the mortality rate and incidence of thyroid cancer by SEER summary stage in men were analyzed, the age‐standardized mortality rate has decreased from 2002 in men and from 2004 in women (Figure 1). After that, the age‐standardized incidence rates of thyroid cancer with distant stage showed decreasing trends from 2010, and the incidence of regional and localized thyroid cancer started to decrease in 2012, respectively (Figure 2).

## 4 DISCUSSION

In South Korea, the incidence of thyroid cancer rapidly increased in both men and women from 1999 to 2008–2009, attenuated from 2008–2009 to 2012, and declined from 2012 to 2016. However, there was no specific change in the anaplastic thyroid cancer incidence during the same time period. In addition, the incidence of localized and regional thyroid cancer started to decrease since 2012, while that of distant thyroid cancer decreased since 2010.

Interestingly, we observed that the thyroid cancer incidence started to decrease simultaneously in 2012–2013 for all age groups except men aged <20 years. This finding suggests that the changing trends in the thyroid cancer incidence were caused by the same factors in all age groups.

Some researchers attributed the decline in thyroid cancer incidence in South Korea to the debates for over diagnosis of thyroid cancer.^4^ Cancer over diagnosis had become a major issue in the United States,^17^, ^18^ but not it is a minor issue in the Korean medical community and society until year 2014. Most Korean doctors who diagnosed and treated thyroid cancer believed that there was no evidence for opposing screening for thyroid cancer.^19^ Although there were similar press issues about thyroid cancer over diagnosis before 2014, those offerings were not big enough to widely affect screening practice for thyroid cancer or result in discussions at related conferences in South Korea. Indeed, previous studies have shown that in the year 2014, thyroid cancer screening was a major issue for the public as well as epidemiologists and thyroid cancer physicians in South Korea.^20^, ^21^ Therefore, considering the incidence of thyroid cancer peaked in 2012 and started to decline in 2013, the debates for over diagnosis of thyroid cancer accelerated the decline of thyroid cancer in South Korea, but it may not be the starting point.

We infer that changes in the examination guidelines for thyroid nodule and thyroid cancer may be the reason why the incidence of thyroid cancer started to decrease from 2012 to 2013, before the societal debate about screening. In 2009, the revised guidelines for the management of thyroid nodules by the American Thyroid Association recommended that only thyroid nodules >1 cm required further examination except for lymphadenopathy or other high‐risk groups.^22^ In 2010, updated guidelines for the management of thyroid nodules were announced in Korea.^23^ This new guideline recommended that fine needle aspiration (FNA) should not be used for thyroid nodule <0.5 cm unless there was evidence of malignancy such as cervical lymph node involvement.^23^ One tertiary hospital reported that there was a decrease in the number and proportion of thyroid cancer <0.5 cm since 2009.^24^ The extent to which the revised guideline affected the reduction in the thyroid cancer incidence is unclear. The positive attitude of physicians regarding the use of guidelines from the academia^25^, ^26^ suggested that these guidelines partly contributed to a decrease in the thyroid cancer incidence.

The stage shift due to thyroid cancer over diagnosis and the decrease in the thyroid cancer reservoir may be another explanation for this decrease of the incidence. First, a stage shift through screening may lead to a reduction in the thyroid cancer incidence.^27^, ^28^ Indeed, the age‐standardized mortality rate started to decrease from 2004, and the incidence of distant thyroid cancer started to decrease since 2010.^29^ Then, the incidences of regional and local thyroid cancer started to decrease since 2012. These sequential changes suggest a stage shift. Second, decreased detection of thyroid cancer in people with thyroid nodules and an annual decrease in thyroid cancer diagnosis/thyroid fine‐needle aspiration (FNA) ratio suggest that the thyroid cancer reservoir has decreased.^30^ Lee et al. showed that thyroid cancer diagnosis/thyroid FNA ratio decreased from 36.5% in 2004 to 25.1% in 2011 using the National Health Insurance database.^30^ Third, our argument could be inferred from the trend of prostate cancer, which is similar to thyroid cancer, in that over diagnosis has been a major issue. Some studies reported that latent prostate cancer decreased after introduction of prostate cancer screening using prostate‐specific antigen (PSA).^31^, ^32^ These study findings suggest that there was a decrease in reservoir of prostate cancer, which represents an over diagnosed cancer, and a similar situation might apply to thyroid cancer. However, it was impossible to precisely confirm the actual decrease in the thyroid cancer reservoir in Koreans; therefore, it was impossible to determine whether this decrease affected the thyroid cancer incidence. Finally, occasional discussions regarding thyroid cancer over diagnosis before 2014 might have partly influenced the practices of thyroid cancer screening.

The present study has several limitations. First, this study is descriptive and does not provide information on the direct cause of the decrease in thyroid cancer. Second, our study did not include the change in the overall reservoirs of thyroid nodules and cancer, changes in the attitude and behavior of physicians regarding thyroid cancer screening such as FNA, and the patients’ participation rate and intentions for thyroid cancer screening. Third, external factors could affect the thyroid cancer incidence simultaneously or interact with each other. Fourth, the proportion of unknown stages was relatively high, and it decreased from 2006 to 2016. Since the unknown stage had a lower 5‐year survival rate than did the local or regional stages, it is likely that this stage contains some proportion of distant thyroid cancer. Nevertheless, our nationwide study provides a clue to the cause of the decrease in the thyroid cancer incidence by describing events before the changing point of thyroid cancer incidence. Ahn et al have only evaluated trends in the number of patients who had surgery for thyroid cancer,^4^ whereas we examined nationwide trends in the thyroid cancer incidence using Joinpoint regression analysis and cancer registration database, which includes almost all cancer patients in South Korea. Moreover, we examined changes in trends by histological types, age groups, and SEER summary stages. The results showed that the starting point of a decreasing trend differed for each stage, with reasons suggested for these trends.

In conclusion, our study showed that the thyroid cancer incidence peaked in 2012 and started to decrease from 2013 in South Korea, before the debates for over diagnosis of thyroid cancer began. Since 2012, the thyroid cancer incidence started to decrease, and this was triggered by changes in guidelines for thyroid nodule examination or a decrease in the thyroid cancer reservoir before the societal controversy regarding over diagnosis. The debates on thyroid cancer over diagnosis may have accelerated the decline in thyroid cancer since 2014.

## CONFLICT OF INTEREST

The authors have no conflicts of interest.

## AUTHOR CONTRIBUTIONS

Young‐Joo Won: study conception and data acquisition, interpretation, and analysis. Chang‐Mo Oh: manuscript writing, study design, data interpretation. Jiwon Lim: data analysis and figure preparation. Yuh Seog Jung, Yeol Kim, Kyu‐Won Jung, and Seri Hong: data acquisition. All authors read and approved the final manuscript for submission.

## ETHICAL STATEMENT

The study was approved by the institutional review board, with exemption of obtaining written informed consent because of the retrospective design of the study.

## Supporting information

Table S1‐S4

## Data Availability

The data that support the findings of this study are available from the Korea Central Cancer Registry upon reasonable request.
