# Supplementary material for: Decreasing trends in thyroid cancer incidence in South Korea: What happened in South Korea?
Source: Cancer Med. 2021 May 12;10(12):4087–96. doi: 10.1002/cam4.3926 (PMC8209587; doi:10.1002/cam4.3926)
Supplement: Supplementary file 1 — Table S1‐S4 [file CAM4-10-4087-s001.docx]

**Supplementary Table 1. Segi’s world standard population**

| **Age group** | **Standard population** |
| --- | --- |
| 0-4 | 12,000 |
| 5-9 | 10,000 |
| 10-14 | 9,000 |
| 15-19 | 9,000 |
| 20-24 | 8,000 |
| 25-29 | 8,000 |
| 30-34 | 6,000 |
| 35-39 | 6,000 |
| 40-44 | 6,000 |
| 45-49 | 6,000 |
| 50-54 | 5,000 |
| 55-59 | 4,000 |
| 60-64 | 4,000 |
| 65-69 | 3,000 |
| 70-74 | 2,000 |
| 75-79 | 1,000 |
| 80-84 | 500 |
| ≥85 | 500 |
| Total | 100,000 |

Reference: Segi M. Cancer mortality for selected sites in 24 countries (1950-1957). Sendai, Japan: Tohoku University School of Medicine; 1960

**Supplementary Table 2. Age-specific incidence rates for thyroid cancer per 100,000 people among Korean men**

| **Age group** | **Year** | | | | | | | | | | | | | | | | | | |
| --- | --- | --- | --- | --- | --- | --- | --- | --- | --- | --- | --- | --- | --- | --- | --- | --- | --- | --- | --- |
|  | **1999** | **2000** | **2001** | **2002** | **2003** | **2004** | **2005** | **2006** | **2007** | **2008** | **2009** | **2010** | **2011** | **2012** | **2013** | **2014** | **2015** | **2016** | **1999-2016** |
| 0-4 | 0.0 | 0.0 | 0.0 | 0.0 | 0.0 | 0.0 | 0.0 | 0.0 | 0.0 | 0.0 | 0.0 | 0.0 | 0.0 | 0.0 | 0.0 | 0.0 | 0.0 | 0.0 | 0.0 |
| 5-9 | 0.0 | 0.1 | 0.1 | 0.0 | 0.1 | 0.1 | 0.0 | 0.1 | 0.1 | 0.1 | 0.1 | 0.1 | 0.0 | 0.4 | 0.3 | 0.1 | 0.2 | 0.2 | 0.1 |
| 10-14 | 0.1 | 0.1 | 0.0 | 0.3 | 0.2 | 0.0 | 0.3 | 0.1 | 0.3 | 0.2 | 0.5 | 0.3 | 0.2 | 0.4 | 0.7 | 0.5 | 0.5 | 0.7 | 0.3 |
| 15-19 | 0.7 | 0.5 | 0.5 | 0.5 | 0.8 | 0.7 | 0.6 | 0.9 | 0.9 | 1.5 | 1.2 | 1.6 | 1.3 | 1.2 | 2.0 | 1.7 | 1.3 | 1.6 | 1.1 |
| 20-24 | 1.1 | 0.7 | 1.0 | 1.1 | 1.4 | 1.5 | 1.9 | 3.0 | 2.7 | 3.2 | 2.9 | 4.1 | 5.1 | 5.9 | 6.3 | 4.4 | 3.7 | 5.2 | 3.0 |
| 25-29 | 1.4 | 1.6 | 1.3 | 1.8 | 2.5 | 4.2 | 4.4 | 4.4 | 7.0 | 9.1 | 9.9 | 12.9 | 14.4 | 18.6 | 20.4 | 15.0 | 14.8 | 17.7 | 8.3 |
| 30-34 | 1.8 | 2.1 | 2.2 | 2.5 | 5.1 | 5.5 | 6.0 | 9.3 | 12.8 | 17.6 | 22.6 | 25.1 | 28.9 | 34.1 | 40.1 | 31.0 | 26.8 | 29.8 | 16.1 |
| 35-39 | 3.1 | 1.8 | 3.2 | 3.4 | 5.7 | 7.6 | 9.2 | 13.1 | 17.7 | 24.1 | 30.8 | 37.1 | 45.4 | 50.5 | 55.3 | 36.9 | 34.1 | 34.5 | 22.5 |
| 40-44 | 2.7 | 2.5 | 3.9 | 4.1 | 6.5 | 10.0 | 12.9 | 14.4 | 22.4 | 27.5 | 34.9 | 43.3 | 47.8 | 55.8 | 56.9 | 41.8 | 38.8 | 39.0 | 26.3 |
| 45-49 | 4.1 | 3.1 | 4.5 | 5.9 | 6.3 | 10.7 | 13.5 | 17.2 | 24.4 | 33.5 | 38.4 | 43.8 | 46.8 | 51.4 | 54.7 | 37.3 | 34.7 | 34.1 | 27.7 |
| 50-54 | 4.0 | 3.0 | 5.2 | 5.4 | 6.9 | 12.1 | 14.4 | 20.7 | 24.2 | 34.2 | 40.4 | 49.9 | 53.4 | 56.9 | 59.7 | 40.1 | 32.0 | 29.5 | 31.4 |
| 55-59 | 5.1 | 5.5 | 5.9 | 6.4 | 7.7 | 12.0 | 17.0 | 20.2 | 23.6 | 33.3 | 37.7 | 45.9 | 52.2 | 55.9 | 52.9 | 43.0 | 32.9 | 33.2 | 30.6 |
| 60-64 | 5.4 | 6.3 | 6.2 | 7.5 | 10.5 | 11.6 | 12.7 | 18.0 | 25.8 | 35.0 | 38.8 | 43.9 | 43.8 | 51.9 | 46.9 | 35.0 | 29.4 | 27.5 | 26.9 |
| 65-69 | 7.4 | 6.4 | 8.3 | 9.4 | 11.8 | 10.4 | 14.8 | 16.8 | 23.4 | 28.9 | 32.6 | 39.2 | 41.8 | 47.6 | 46.7 | 32.1 | 25.4 | 25.5 | 25.8 |
| 70-74 | 8.0 | 5.8 | 9.8 | 8.2 | 12.2 | 11.6 | 14.7 | 15.6 | 17.7 | 21.7 | 24.7 | 28.6 | 27.5 | 33.2 | 32.5 | 23.9 | 20.5 | 19.4 | 20.8 |
| 75-79 | 8.0 | 10.2 | 8.0 | 6.9 | 6.7 | 8.1 | 15.2 | 12.8 | 15.8 | 17.6 | 17.6 | 18.9 | 22.2 | 25.8 | 23.8 | 18.8 | 12.3 | 13.9 | 15.9 |
| 80-84 | 3.3 | 7.3 | 8.8 | 11.0 | 10.1 | 11.1 | 4.6 | 13.2 | 6.4 | 13.5 | 17.7 | 13.5 | 13.7 | 20.1 | 15.6 | 17.1 | 11.9 | 14.7 | 12.9 |
| ≥85 | 4.8 | 9.3 | 6.5 | 12.2 | 15.2 | 12.5 | 6.6 | 7.6 | 13.9 | 6.3 | 9.3 | 11.0 | 5.2 | 14.7 | 9.1 | 6.6 | 10.7 | 5.7 | 9.2 |
| Total | 2.2 | 2.0 | 2.6 | 3.0 | 4.3 | 5.8 | 7.3 | 9.5 | 13.0 | 17.5 | 21.1 | 25.4 | 28.3 | 32.4 | 33.8 | 24.6 | 21.3 | 21.7 | 15.5 |

The incidence rates are calculated per 100,000 people.

**Supplementary Table 3. Age-specific incidence rates for thyroid cancer per 100,000 people among Korean women**

| **Age group** | **Year** | | | | | | | | | | | | | | | | | | |
| --- | --- | --- | --- | --- | --- | --- | --- | --- | --- | --- | --- | --- | --- | --- | --- | --- | --- | --- | --- |
|  | **1999** | **2000** | **2001** | **2002** | **2003** | **2004** | **2005** | **2006** | **2007** | **2008** | **2009** | **2010** | **2011** | **2012** | **2013** | **2014** | **2015** | **2016** | **1999-2016** |
| 0-4 | 0.0 | 0.0 | 0.1 | 0.0 | 0.0 | 0.0 | 0.0 | 0.0 | 0.0 | 0.0 | 0.0 | 0.0 | 0.0 | 0.0 | 0.0 | 0.0 | 0.2 | 0.0 | 0.0 |
| 5-9 | 0.2 | 0.2 | 0.1 | 0.1 | 0.1 | 0.0 | 0.1 | 0.1 | 0.0 | 0.2 | 0.1 | 0.5 | 0.3 | 0.2 | 0.2 | 0.1 | 0.3 | 0.1 | 0.2 |
| 10-14 | 0.8 | 0.9 | 0.9 | 0.5 | 0.4 | 0.8 | 0.7 | 0.8 | 1.1 | 1.6 | 0.9 | 1.5 | 1.8 | 2.2 | 1.8 | 1.6 | 2.0 | 1.0 | 1.2 |
| 15-19 | 2.2 | 2.7 | 2.5 | 3.5 | 4.4 | 5.2 | 4.9 | 4.5 | 5.5 | 5.8 | 6.7 | 7.5 | 8.2 | 7.5 | 9.3 | 9.1 | 7.7 | 8.9 | 5.8 |
| 20-24 | 6.5 | 5.6 | 8.0 | 8.1 | 11.0 | 14.1 | 13.2 | 16.5 | 17.1 | 22.2 | 21.0 | 26.9 | 29.9 | 30.3 | 32.4 | 27.8 | 23.8 | 26.4 | 18.1 |
| 25-29 | 9.9 | 10.3 | 13.6 | 14.8 | 19.5 | 24.1 | 27.4 | 30.7 | 38.2 | 51.3 | 61.3 | 70.1 | 75.0 | 81.7 | 85.1 | 68.4 | 57.3 | 67.3 | 42.2 |
| 30-34 | 11.7 | 11.0 | 16.0 | 19.6 | 27.9 | 36.5 | 43.3 | 56.1 | 69.1 | 89.2 | 108.2 | 124.8 | 136.1 | 145.5 | 146.1 | 106.2 | 90.7 | 95.0 | 71.2 |
| 35-39 | 15.3 | 15.1 | 20.9 | 24.1 | 35.4 | 48.8 | 57.9 | 72.7 | 91.4 | 120.8 | 148.8 | 164.0 | 182.4 | 194.8 | 183.5 | 140.2 | 111.9 | 119.3 | 96.2 |
| 40-44 | 18.2 | 19.3 | 27.6 | 34.5 | 46.7 | 67.6 | 78.6 | 95.2 | 128.3 | 156.0 | 181.3 | 199.1 | 220.9 | 227.0 | 228.5 | 166.0 | 131.2 | 139.7 | 122.3 |
| 45-49 | 23.3 | 23.2 | 30.3 | 39.9 | 57.4 | 81.3 | 97.2 | 127.5 | 160.5 | 191.4 | 218.8 | 236.8 | 255.0 | 260.2 | 237.4 | 164.5 | 128.3 | 128.6 | 145.4 |
| 50-54 | 27.0 | 27.3 | 33.6 | 44.1 | 57.1 | 82.7 | 102.6 | 128.7 | 168.4 | 208.7 | 236.7 | 259.5 | 287.6 | 301.1 | 273.3 | 187.7 | 144.0 | 142.2 | 169.3 |
| 55-59 | 26.7 | 24.9 | 32.0 | 44.2 | 57.5 | 77.4 | 93.5 | 117.0 | 146.7 | 189.8 | 209.8 | 228.4 | 261.7 | 274.5 | 236.9 | 167.6 | 127.0 | 123.7 | 146.2 |
| 60-64 | 24.4 | 22.4 | 28.1 | 30.5 | 46.7 | 61.0 | 76.9 | 90.5 | 129.7 | 153.7 | 187.4 | 200.7 | 224.8 | 244.6 | 212.7 | 140.0 | 113.3 | 108.0 | 120.3 |
| 65-69 | 21.3 | 18.6 | 25.4 | 28.9 | 35.2 | 46.1 | 62.6 | 67.1 | 91.4 | 110.1 | 133.4 | 147.2 | 157.2 | 180.0 | 160.2 | 114.6 | 84.8 | 88.6 | 91.9 |
| 70-74 | 17.4 | 15.0 | 19.6 | 23.8 | 25.5 | 31.2 | 37.4 | 44.4 | 54.1 | 73.3 | 90.7 | 89.0 | 106.6 | 110.8 | 108.4 | 75.5 | 60.3 | 58.2 | 63.5 |
| 75-79 | 16.1 | 14.7 | 15.5 | 20.2 | 19.2 | 24.2 | 30.4 | 29.9 | 32.7 | 39.3 | 50.8 | 52.9 | 62.0 | 65.7 | 58.4 | 40.4 | 35.0 | 41.9 | 39.3 |
| 80-84 | 11.1 | 13.1 | 14.3 | 14.8 | 19.1 | 13.6 | 17.6 | 14.5 | 26.4 | 28.3 | 25.4 | 33.2 | 30.8 | 34.7 | 28.2 | 22.0 | 20.9 | 23.2 | 23.1 |
| ≥85 | 12.2 | 13.2 | 8.0 | 13.3 | 9.1 | 11.0 | 19.3 | 14.5 | 13.4 | 22.2 | 15.4 | 12.9 | 18.9 | 17.2 | 17.1 | 15.1 | 12.1 | 13.2 | 14.7 |
| Total | 12.0 | 11.9 | 15.9 | 19.7 | 27.0 | 37.3 | 45.4 | 56.7 | 73.9 | 93.3 | 110.0 | 122.2 | 136.6 | 145.0 | 136.2 | 98.0 | 77.9 | 80.2 | 73.1 |

The incidence rates are calculated per 100,000 people.

**Supplementary Table 4. Age-standardized incidence rates for thyroid cancer per 100,000 people by sex in South Korea from 1999-2001 to 2014-2016**

| **Categories** | **1999–2001** | **2002–2004** | **2005–2007** | **2008–2010** | **2011–2013** | **2014–2016** |
| --- | --- | --- | --- | --- | --- | --- |
|  |  |  |  |  |  |  |
| **Men** | 2.16 | 3.76 | 7.86 | 15.90 | 22.67 | 16.08 |
| **Age at Diagnosis** |  | | | | | |
| <20 years | 0.16 | 0.19 | 0.24 | 0.43 | 0.50 | 0.51 |
| 20-44 years | 1.95 | 3.94 | 8.75 | 18.74 | 29.91 | 23.18 |
| 45-54 years | 4.00 | 7.95 | 19.22 | 40.04 | 53.59 | 34.71 |
| 55-64 years | 5.73 | 9.34 | 19.58 | 39.31 | 50.62 | 33.32 |
| ≥65 years | 7.63 | 10.36 | 15.87 | 25.87 | 33.66 | 21.60 |
| **Histology type**^a^ |  | | | | | |
| Papillary | 1.58 | 3.12 | 6.85 | 14.84 | 21.72 | 15.30 |
| Follicular | 0.19 | 0.24 | 0.32 | 0.37 | 0.44 | 0.34 |
| Medullary | 0.06 | 0.09 | 0.11 | 0.13 | 0.17 | 0.11 |
| Anaplastic | 0.07 | 0.05 | 0.06 | 0.06 | 0.06 | 0.06 |
| Others | 0.26 | 0.26 | 0.53 | 0.51 | 0.26 | 0.26 |
| **SEER summary Stage**^b^ |  | | | | | |
| Localized | - | - | 2.85 | 5.77 | 8.20 | 5.27 |
| Regional | - | - | 3.56 | 8.20 | 12.78 | 9.42 |
| Distant | - | - | 0.22 | 0.26 | 0.24 | 0.16 |
| Unknown | 2.07 | 3.65 | 1.23 | 1.67 | 1.44 | 1.23 |
| **Women** | 11.27 | 22.67 | 45.03 | 79.99 | 99.94 | 61.58 |
| **Age at Diagnosis** |  | | | | | |
| <20 years | 0.80 | 1.14 | 1.33 | 1.86 | 2.36 | 2.34 |
| 20-44 years | 13.37 | 27.23 | 51.89 | 95.78 | 124.12 | 86.04 |
| 45-54 years | 27.41 | 61.00 | 131.83 | 224.81 | 267.39 | 148.43 |
| 55-64 years | 26.41 | 53.10 | 109.19 | 195.63 | 242.23 | 129.15 |
| ≥65 years | 18.24 | 28.49 | 51.73 | 90.07 | 114.42 | 67.61 |
| **Histology type**^a^ |  | | | | | |
| Papillary | 9.64 | 20.48 | 41.43 | 76.32 | 97.34 | 59.41 |
| Follicular | 0.81 | 1.04 | 1.10 | 1.25 | 1.38 | 1.03 |
| Medullary | 0.13 | 0.17 | 0.21 | 0.29 | 0.31 | 0.17 |
| Anaplastic | 0.10 | 0.08 | 0.07 | 0.10 | 0.08 | 0.07 |
| Others | 0.59 | 0.89 | 2.23 | 2.03 | 0.84 | 0.90 |
| **SEER summary Stage**^b^ |  | | | | | |
| Localized | - | - | 19.41 | 34.72 | 44.19 | 24.85 |
| Regional | - | - | 18.29 | 37.20 | 49.40 | 32.13 |
| Distant | - | - | 0.49 | 0.65 | 0.48 | 0.30 |
| Unknown | - | - | 6.83 | 7.42 | 5.88 | 4.29 |

The age-standardized incidence rates are calculated as incidence cases per 100,000 people using Segi’s world standard population.

^a^The histological subtypes of thyroid cancer were classified as papillary thyroid carcinoma, follicular thyroid carcinoma, medullary thyroid carcinoma, anaplastic thyroid carcinoma and others according to the International Classification of Diseases for Oncology, 3rd edition.

^b^SEER summary stage was classified into localized stage, regional stage, distant stage and unknown stage and it has been collected nationally since 2006.
